# Original Training for Laparoscopic Surgery by Making an Origami Paper Crane

**DOI:** 10.7759/cureus.62098

**Published:** 2024-06-10

**Authors:** Yusuke Noda, Shuzo Hamamoto, Takumi Shiraki, Takuya Sakata, Nami Tomiyama, Taku Naiki, Daisuke Matsumoto, Tomoki Okada, Hiroki Kubota, Takahiro Yasui

**Affiliations:** 1 Urology, Anjo Kosei Hospital, Anjo, JPN; 2 Urology, Nagoya City University Graduate School of Medical Sciences, Nagoya, JPN; 3 General Surgery, Hamamatsu University School of Medicine, Hamamatsu, JPN; 4 Urology, Kainan Hospital Aichi Prefectural Welfare Federation of Agricultural Cooperatives, Yatomi, JPN

**Keywords:** origami, paper crane, learning curve, laparoscopic origami training, surgical training, laparoscopic surgery

## Abstract

Introduction: The training box is an effective tool used by surgical trainees. Suturing training is the common method of practicing laparoscopic surgery; however, the cost of needles and threads for long-term practice remains a problem. In this study, we incorporated the original Japanese training for laparoscopic surgery by making an origami paper crane (laparoscopic origami training (LOT)) and evaluated its effect on the clinical results as a long-term practice.

Methods: LOT was performed using a single 7.5 × 7.5 cm origami paper in the training box of laparoscopic surgery. In the bench-top study, the total time required to make one paper crane was measured and evaluated, and a self-efficacy questionnaire was designed to analyze the efficacy of LOT. In clinical practice, we retrospectively compared two resident groups, one that had previously trained on LOT (trained group) and the other that did not (less-trained group), by analyzing the pneumoperitoneum time (PT) for 10 cases.

Results: After making paper cranes in approximately 100 cases, the making time was reduced to approximately 10 min. Long-term results analyzing up to 1500 cases revealed that in addition to shortening the time required to make a paper crane, the shape of the crane also improved. Consequently, the median PT was significantly shorter in the trained group than in the less-trained group (129.0 (62-287) versus 208.5 (127-343) min; p<0.001).

Conclusion: LOT contributed to introducing safe laparoscopic surgery to residents and improved their laparoscopic outcomes. We believe that this is a useful practice methodology that can be recommended to general physicians who wish to practice laparoscopic surgeries.

## Introduction

The introduction of robotic surgery has led to a decrease in the importance of laparoscopic surgery. However, laparoscopic surgery is still the most commonly used technique for radical nephrectomy and nephroureterectomy because of its advantages, including decreased cost and operative time [1]. Nevertheless, there is a significant learning curve with laparoscopic surgery, and the widespread introduction of this technique requires careful monitoring and supervision [2,3].

Skill acquisition outside the operating room has become an integral and important adjunct to surgical training. Training using a training box for laparoscopic surgery is one of the surgical training methods [4]. To date, few residents are satisfied with such training [5,6] because the most commonly practiced technique in the training box is generally suturing. The practice of suturing poses challenges in terms of long-term training. It is difficult for residents to maintain their motivation with few methods to evaluate the efficacy; moreover, suturing training requires many needle threads.

Based on this background, we established a low-cost, long-term continuable training by making an origami paper crane, i.e., laparoscopic origami training (LOT), in which a flat square sheet of paper is transformed into a paper crane. Origami is the art of paper folding and is often associated with the Japanese culture. Folding the paper carefully results in a functional representation of an object shape, and the paper crane is one of the most popular creations. This study aimed to evaluate the efficiency of LOT and evaluate its effect on clinical results.

## Materials and methods

Ethics statement

The study was conducted in accordance with the Declaration of Helsinki and approved by the Institutional Review Board at Nagoya City University (approval number: 60-19-196).

Training instruments

**Figure 1:**
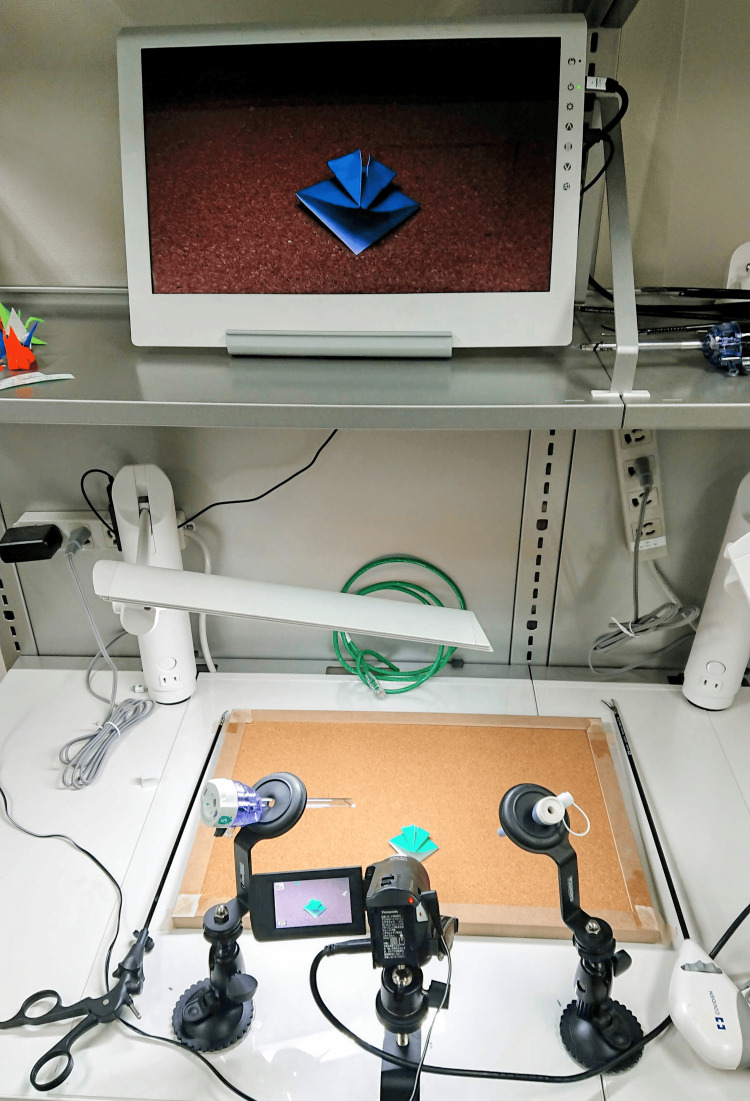
Training box of laparoscopic origami training The instruments include a pair of LapaSta® training needles, a mobile display monitor, and a video camera connected with micro-HDMI cables.

Our LOT instruments are presented in Figure 1. They included a pair of LapaSta® training needle holders (Japan Polymer Technology, Tokyo, Japan) that can fix the surgical trocar, a mobile display monitor (GeChic On-Lap 2501; GeChic, Taichung City, Taiwan), and a video camera (HD Videocamera V480MS; Panasonic Corporation, Osaka, Japan); these instruments were connected with micro-HDMI cables. It costs approximately $650 to set up the training instruments. There were no restrictions on the forceps used, but we mainly used the Maryland-type Ligasure™ forceps (Medtronic Japan, Tokyo, Japan) in the right hand and a Microline Renew™ (AMCO Inc., Tokyo, Japan) in the left hand. Additionally, a corkboard, other cutting mats, and quilted fabric carpet pads were provided.

Laparoscopic origami training

**Figure 2:**
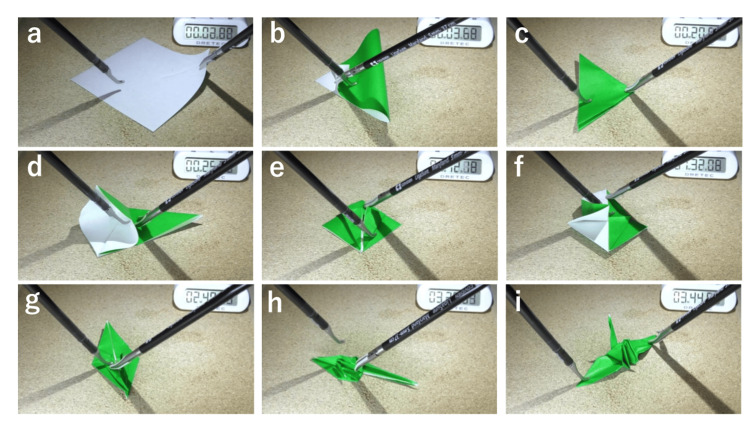
Process of making the origami paper crane with laparoscopic forceps A 7.5 × 7.5 cm piece of origami paper was provided. Maryland-type Ligasure™ forceps in the right hand and the Microline Renew™ in the left hand were used for training. (a) original setting; (i) completed origami paper crane.

The LOT methods are presented in Figure 2 and the supplemental video (https://drive.google.com/file/d/1-AWu37xDwEHTDSmfManDrLzw3TvJDw7s/view?usp=share_link). Surgeons started with a 7.5 × 7.5 cm piece of origami paper (Figure 2A). They folded the paper in half by taking the top corner and folding it to the bottom corner. The crease should run from the left corner tip to the right, as shown in Figure 2B. Then, they folded the triangle in half by taking the right corner and folding it to the left side (Figure 2C). They grasped the top flap and opened it, creasing the left and right sides to fold the top right corner to the bottom corner. Next, they turned the paper over and followed the same steps on the other side (Figure 2D). They took both sides of the top layer, folded them such that they met in the middle, and unfolded them. This step prepared them for the next step, as depicted in Figure 2E. Subsequently, they opened the flap upwards, folded the left and right sides inward, flipped the paper over, and repeated these steps (Figure 2F). Urologists grasped the upper layer of both sides and folded the lower parts to the center. The paper was turned over so that the same step could be repeated on the other side. The right flap was folded over to the left side, and the whole piece of paper was again turned over to repeat the same step on the other side. They grasped the bottom flap and folded it over the top; then, they flipped the paper over and repeated the process on the other side. Next, the top layer of the right flap was folded over to the left side. The paper was flipped over to do the same process on the other side (Figure 2G). Urologists took one of the pieces that they pulled apart, slightly opened the top corner, and bent a portion of it downward to form the head (Figure 2H). After the portion of the paper was bent down, the sides of the head were creased so that the piece would stay bent. The wings were bent down at a 90° angle. Finally, they created a paper crane with forceps (Figure 2I). The total time required to make one paper crane was measured and evaluated. The supplemental video can be accessed at the link https://www.youtube.com/watch?v=Mh6CnYLzYvM.

Clinical outcomes

We retrospectively analyzed the pneumoperitoneum time (PT) of the initial 10 cases of laparoscopic nephrectomy (LRN) or laparoscopic nephroureterectomy (LRNU) that eight urologists performed at the Nagoya City University Hospital, Nagoya, Japan, between January 2010 and December 2021. The participants were divided into two groups: a group of four urologists who had undergone laparoscopic training by making an origami paper crane >100 times (trained group) and another group of four urologists who had never undergone such training (less-trained group). The participants in the trained group continued LOT simultaneously with performing the surgeries. All laparoscopic surgeries were performed using four trocars via the retroperitoneal approach, with the patient in the lateral kidney position. No arbitrary selection was made based on the difficulty of the case, and each physician performed the procedure on the patient assigned to them in the outpatient clinic. We retrospectively compared the surgical outcomes between the trained and less-trained groups to determine the real-world clinical impact of LOT.

Self-efficacy questionnaire

A self-efficacy questionnaire was designed to survey the status of LOT (supplementary methods). A Google form was used to design the questionnaire and gather data from medical students, residents, and physicians worldwide who had experienced LOT.

Statistical analysis

The PT was analyzed between the two groups. All data were analyzed using descriptive statistics and are presented as percentages. Continuous variables were compared using the Mann-Whitney U-test. A p-value <0.05 was considered statistically significant. All statistical analyses were performed using EZR for R (The R Project for Statistical Computing, Vienna, Austria) [7].

## Results

Laparoscopic training data

**Figure 3:**
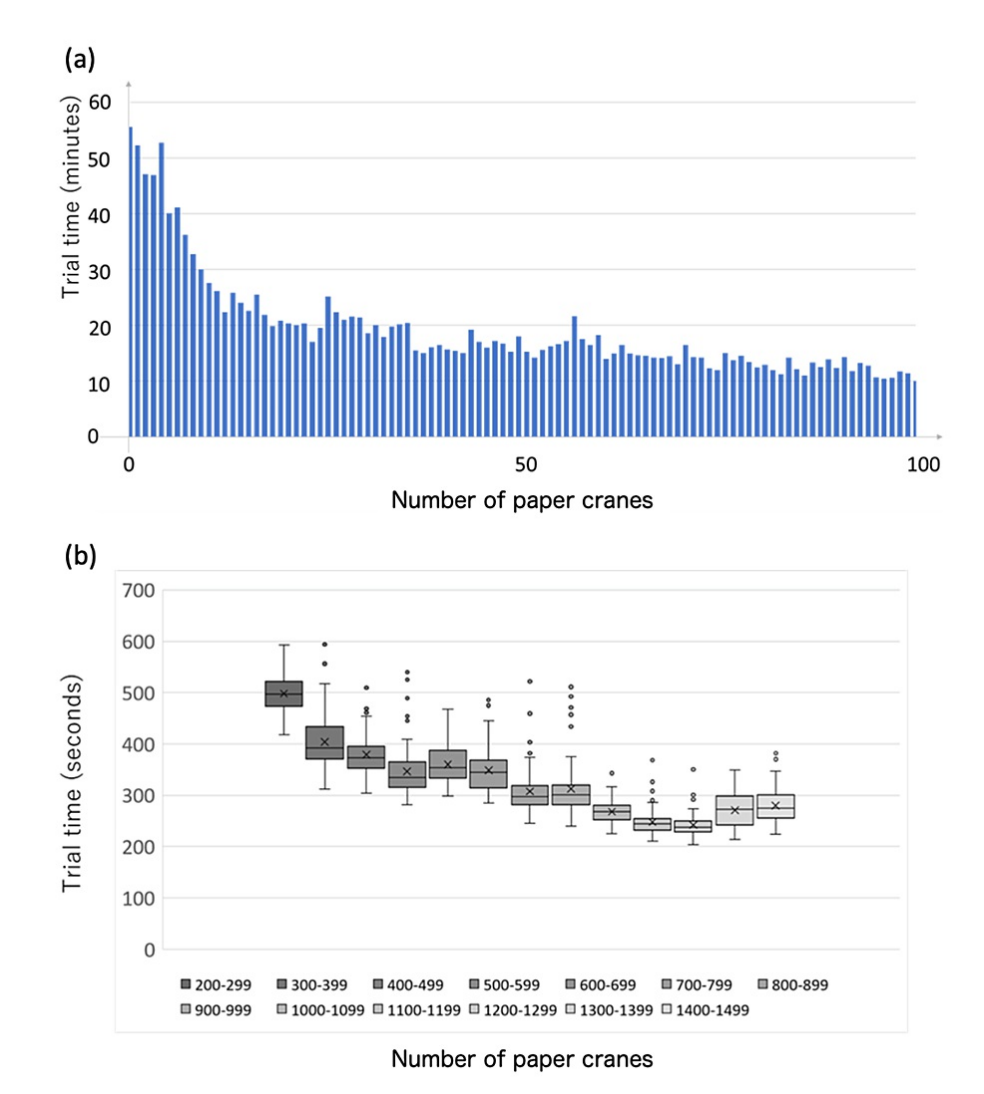
Time of laparoscopic origami training (a) Initial 100 cases and (b) times of 100 cases displayed in a box-and-whisker plot. The shortest time is 204 s.

**Figure 4:**
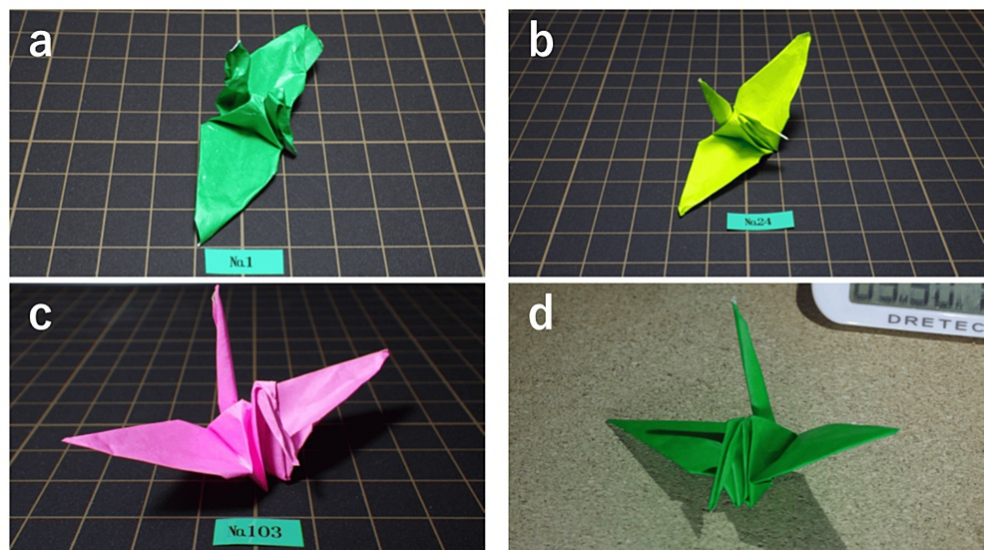
Paper cranes of select cases (a) 1, (b) 24, (c) 103, and (d) 1542. With each case, the shape of the paper crane improved. A 7.5 × 7.5 cm paper was used. With each case, the design was perfected.

The total time to make one paper crane is presented in Figures 3A, 3B. Figure 3A shows the outcomes of T.S. making up to 100 cranes. Figure 3B shows the change in time taken by Y.N. to make 200-1500 cranes. Initially, it took approximately 1 hour to complete the training; however, the duration gradually decreased. After making a paper crane in nearly 100 cases, it became possible for the urologists to make paper cranes in approximately 10 min (Figure 3A). Even in the long-term results of up to 1500 cases, the time required to make a paper crane became increasingly shorter (Figure 3B). The shortest time was 3 min and 24 seconds (204 seconds). Figures 4A-4D show the shape of the paper cranes. The shape became increasingly well-defined with each case.

Clinical outcomes

To determine the impact of LOT on real-world clinical practice, we retrospectively compared the surgical outcomes in the trained and non-trained groups. There were no significant differences in the surgeons’ age and sex and patients’ age, sex, and body mass index between the trained and less-trained groups. The PT was significantly shorter in the trained group than in the less-trained group (129.0 (62-287) versus 208.5 (127-343) min, p<0.001). The surgeons’ and patients’ characteristics are presented in Table 1.

**Table 1:** Results of the self-efficacy questionnaire

Variable	n	%
Age (years)		
20-29	38	35.8
30-39	48	45.3
40-49	15	14.2
50-59	5	4.7
Sex		
Male	92	86.8
Female	14	13.2
Specialty		
Resident	8	7.5
Urology	33	31.1
Surgery	43	40.6
Gynecology	18	17.0
Veterinarian	4	3.8
Certification of laparoscopic surgery		
Yes	14	13.2
No	92	86.8
Number of laparoscopic surgeries performed to date (cases)		
0	12	11.3
1-9	19	17.9
10-29	15	14.2
30-49	18	17.0
50-	42	39.7
Number of origami cranes folded completely thus far (cases)		
0	7	6.6
1-49	27	25.5
50-99	13	12.3
100-299	23	21.7
300-999	15	14.2
1000-	21	19.8
Number of hours per month spent on laparoscopic origami training (hours)		
<1	14	13.2
1-4	34	32.1
5-10	24	22.6
11-20	20	18.9
20-	13	12.3
Frequency of training per week (number of times)		
<1	15	14.2
1–2	16	15.1
3-4	31	29.2
5-6	14	13.2
7	30	28.3
Continuation of laparoscopic origami training		
Yes	79	74.5
No	27	25.5
Usefulness of self-appraisal as a tool for learning		
Strongly agree	40	37.7
Agree	49	46.2
Undecided	8	7.5
Disagree	4	3.8
Strongly disagree	5	4.7

Self-efficacy questionnaire

The results of the self-efficacy questionnaire are presented in Table 2. A total of 106 physicians who had performed LOT responded to the questionnaire. The respondents comprised of 92 (86.8%) men and 14 (13.2%) women. Eighty-six (81.1%) respondents were 20-39 years of age. The participants comprised 8 (7.5%) residents and 98 (92.5%) attending physicians. Fourteen (13.2%) respondents were certified in laparoscopic surgery in their own department. Twelve (11.3%) respondents had never performed laparoscopic surgery, whereas 42 (39.7%) physicians had experience with more than 50 cases of laparoscopic surgeries. Regarding LOT, 59 (55.6%) respondents had completely folded more than 100 origami cranes using laparoscopy. Regarding continuity, 57 (53.8%) respondents trained at least 5 hours per month, while 75 (70.8%) trained at least three times per week. In addition, 79 (74.5%) respondents were still continuing this training, and 30 (28.3%) trained almost every day. In total, 89 (83.9%) respondents perceived the training to be useful and effective for learning the laparoscopic technique.

**Table 2:** Surgeons’ and patients’ characteristics BMI: body mass index The chi-square test was used for sex differences and the Mann-Whitney U test for age, BMI, and pneumoperitoneum time.

	Trained group	Less-trained group	p-value
Surgeon characteristics	n = 4	n = 4	
Age, years (average)	31.8	37.8	0.460
Sex, n (%) male	2 (50)	4 (100)	0.0286
Sex, n (%) female	2 (50)	0 (0)	0.0286
Patient characteristics	n = 40	n = 40	
Age, years (range)	72.5 (65.5-77.25)	72 (58.0-79.0)	0.787
Sex, n (%) male	23 (57.5)	28 (70)	0.292
Sex, n (%) female	17 (42.5)	12 (30)	0.292
Median BMI, kg/m^2^ (range)	22.3 (20.38-24.73)	23.16 (21.68-24.69)	0.352
Median pneumoperitoneum time, min (range)	129.0 (62-287)	208.5 (127-343)	<0.001

## Discussion

In this study, we first developed the Japanese original training for laparoscopic surgery by making an origami paper crane, which has been accepted by many physicians as an effective and sustainable training method. Laparoscopic training allows trainees to facilitate learning transfer to real operations, shorten the learning curve, and acquire motor skills and necessary structural safety in the surgical environment [8,9]. Skill training includes the dry laboratory, animal laboratory, and augmented reality simulators of the suture technique, and each training has its own advantages. The key considerations in skill training appear to be effectiveness, continuity, and cost.

Surgical training applying a subtle external force at the tip of the forceps is similar to surgical dissection or retraction movement. The dry and animal laboratories offer the advantages of real strength and the haptic touch as there is interaction with actual instruments. Teaching basic laparoscopic skills to novices before a more complex laparoscopic task produces substantial cost savings [10]. However, virtual simulators are purely virtual instruments that control an integrated mechanism through appropriate sensors. The simulating software can reproduce scenarios and platforms with different procedures, allowing progressive difficulty levels. Nevertheless, it is difficult to practice power application, as it is necessary to adjust the power to a more delicate level than that used in suture training [11,12]. Laparoscopic skills require knob control, torque, force adjustment, and spatial perception [13]. Yoshida et al. reported that the key to safe laparoscopic surgery is to apply force most efficiently in the vertical direction to create an initial dissection point and then change to the horizontal direction to separate the target organ from surrounding tissues [14]. In this original training, coordinated hand movement is necessary to make the paper crane. The paper must be held in one hand and advanced with the other hand while considering the distribution of vertical and horizontal forces. This is similar to the real surgical procedure. In the field of urology, it is possible to practice using forceps in LRN and LRNU. It is difficult to use forceps when practicing suturing training.

A certain amount of long-term training is necessary to improve laparoscopic surgical skills. Trainees may benefit from ongoing training to maintain optimal proficiency [15,16]. However, only a few reports of long-term training, similar to the present report, exist. Many trainees could not complete the entire curriculum as specified. Nonetheless, this training kept trainees motivated, as it allowed them to set the target time, and they were able to shorten their training time after 800 cases. The questionnaire revealed that many surgeons were involved in LOT and appreciated the practice. Moreover, more than 50% of physicians spent at least 5 hours per month on this training and completely folded more than 100 origami cranes using laparoscopy. The result of technical improvement was reflected in the improved design of their paper crane. It takes a certain amount of practice time to improve, and training can continue without boredom. The training described herein can also be used for warm-up. The practice of preoperative warm-up training seems to benefit surgical performance, even for inexperienced laparoscopic surgeons [17]. Practice in the training box is also useful in robot-assisted surgery, and such practice may also help improve surgeons’ skills in robot-assisted surgery [18].

Compared with open surgery, laparoscopic surgery allows surgeons to view surgical images, making it easier to learn procedures [19]. In this study, the practitioners also worked on understanding the surgical procedure with videos and textbooks. We believe that this training enabled surgeons to perform LRN and LRNU without complications within a reasonable operative duration. It is more important to finish the operation safely and without complications rather than quickly, but in LRNU, prolonged PT is an independent risk factor for intravesical recurrence [20]. Thus, this training can improve not only the operative time and complications but also the patient’s prognosis.

Another feature of LOT is its low cost and easy accessibility, which are important in continuous training [21,22]. The initial setup cost of LOT was approximately $650. The cost of this training is very low compared with that of standard laparoscopic training, which consists of six scopes and a monitor that costs $2850.00 [23]. The running cost was also low. Generally, to continue long-term laparoscopic suturing training, many needles are required, which is costly. However, LOT only required a 7.5 × 7.5 cm piece of paper. Such low-cost methods may enable long-term training.

The present study has certain methodological limitations. First, its retrospective design and small sample size limited the evaluation of the association between the efficacy of LOT and surgical outcomes. In addition, the surgeons’ supervisors, procedures, and timing of surgical procedures were not completely standardized. However, if the number of practitioners who perform this training increases in the future, it may be possible to report findings with a high level of evidence [24]. According to the questionnaire, at least 100 physicians are currently working on LOT. In addition, several physicians who are skilled in laparoscopic surgery have also undergone this training and believe that this practice is worthwhile. It is difficult to quantify the technique of laparoscopic surgery, but there are various reports to validate it. It is desirable to verify the progress of operative technology with the help of this training by evaluating force, movement, and acceleration, which apply to external force [25].

Second, the introduction of robotic surgery has led to a decrease in the importance of laparoscopic surgery. LOT may, therefore, lose relevance in the future due to the widespread adoption of robotic surgery. However, it was reported that a trainee’s baseline laparoscopic skills correlate with certain baseline robotic skills. Better baseline performance on an advanced but not basic laparoscopic and robotic skill task may correlate with a shorter learning curve for basic robotic skills [18]. Hence, LOT can also provide good practice for robotic surgery.

Third, origami training and actual surgical procedures such as nephrectomy are considerably different. Origami training does not simulate bleeding or the structure and texture of tissues. Moreover, although the Japanese are familiar with origami, those who do not know how to fold origami or paper cranes may find this form of training challenging. Nevertheless, the questionnaire included non-Japanese physicians, and there were physicians who started LOT without knowing how to fold origami cranes and still found it useful as training for actual surgery. Furthermore, this training may be especially useful for physicians new to laparoscopic surgery because it allows them to learn basic movement skills such as grasping, dissection, pushing maneuvers, coordinated movements of left and right forceps, and hand-eye coordination skills. Although a certain learning curve is required to acquire these skills, some physicians find LOT to be a low-cost method, even for long-term practice, and the long learning curve allows them to continue without losing interest. These improvements in basic manipulation skills may be useful for acquiring the necessary skills for actual surgical procedures such as nephrectomy.

LOT is becoming more widespread, especially in Japan, where Hiraki et al. and Takeda et al. have reported its usefulness [26,27]. They evaluated conventional training methods and their effectiveness in improving laparoscopic manipulation techniques. Our recommendation for this training is based on the subjective opinions of the physicians who have been trained; therefore, further evidence is necessary before this technique can be recommended to more physicians. In this study, we compared clinical outcomes to assess the usefulness of this training in preparation for an actual surgical procedure. In the future, each department can examine the potential usefulness of LOT as a surgical training tool. Nevertheless, training for surgery is different from treatment selection in medical practice, and we feel that physicians should incorporate surgical training that suits them throughout the preparation, study, and practice for surgery, regardless of the level of evidence.

## Conclusions

In this study, we developed a Japanese LOT in which surgeons made an origami paper crane. This LOT led to safe laparoscopic surgery for novice residents and improved their learning curve. Additionally, this cost-effective and motivational training enabled residents to continue training for a long period. We believe that this is a useful practice method that can be recommended to general physicians who want to practice laparoscopic surgeries.

As residents trained more, the time for making an origami crane decreased significantly with an improvement in the quality and structure of the crane.
